# Learning from an equitable, data‐informed response to COVID‐19: Translating knowledge into future action and preparation

**DOI:** 10.1002/lrh2.10369

**Published:** 2023-04-13

**Authors:** Morgen Stanzler, Johanna Figueroa, Andrew F. Beck, Marianne E. McPherson, Steve Miff, Heidi Penix, Jessica Little, Bhargavi Sampath, Pierre Barker, David M. Hartley

**Affiliations:** ^1^ Institute for Healthcare Improvement Boston Massachusetts USA; ^2^ Cincinnati Children's Hospital Medical Center Cincinnati Ohio USA; ^3^ University of Cincinnati College of Medicine Cincinnati Ohio USA; ^4^ Parkland Center for Clinical Innovation (PCCI) Dallas Texas USA; ^5^ Civitas Networks for Health Portland Maine USA

**Keywords:** data, equity, learning systems, trust

## Abstract

**Introduction:**

The COVID‐19 pandemic revealed numerous barriers to effectively managing public health crises, including difficulties in using publicly available, community‐level data to create learning systems in support of local public health decision responses. Early in the COVID‐19 pandemic, a group of health care partners began meeting to learn from their collective experiences. We identified key tools and processes for using data and learning system structures to drive equitable public health decision making throughout different phases of the pandemic.

**Methods:**

In fall of 2021, the team developed an initial theory of change directed at achieving herd immunity for COVID‐19. The theoretical drivers were explored qualitatively through a series of nine 45‐min telephonic interviews conducted with 16 public health and community leaders across the United States. Interview responses were analyzed into key themes to inform potential future practices, tools, and systems. In addition to the interviews, partners in Dallas and Cincinnati reflected on their own COVID‐19 experiences.

**Results:**

Interview responses fell broadly into four themes that contribute to effective, community driven responses to COVID‐19: real‐time, accessible data that are mindful of the tension between community transparency and individual privacy; a continued fostering of public trust; adaptable infrastructures and systems; and creating cohesive community coalitions with shared alignment and goals. These themes and partner experiences helped us revise our preliminary theory of change around the importance of community collaboration and trust building and also helped refine the development of the Community Protection Dashboard tool.

**Conclusions:**

There was broad agreement amongst public health and community leaders about the key elements of the data and learning systems required to manage public health responses to COVID‐19. These findings may be informative for guiding the use of data and learning in the management of future public health crises or population health initiatives.

## 1 INTRODUCTION

More than 2.5 years into the COVID‐19 pandemic, the Centers for Disease Control and Prevention (CDC) announced a significant reorganization aimed at refocusing itself on public health needs, responding more rapidly to emergencies such as outbreaks and emergent epidemics, and providing local and state health authorities and the public alike better information to drive awareness and decision making.^1^ Indeed, the pandemic has magnified the need, from national to “hyper‐local” levels, for better tools to guide the responses required in rapidly evolving, uncertain circumstances. To achieve this informed responsiveness, individuals, communities, and organizations need better ways to access, synthesize, learn from, and incorporate data into decisions that help them manage situations like vaccine distribution and delivery,^2^ and phases of the COVID‐19 pandemic now and into the future.^3^

The large variation in the speed and intensity of COVID‐19 infection^4^ across different communities highlights the urgent and ongoing need for leaders across health care, public health, and the government to have real‐time, local data to drive decision‐making. Moreover, these data needed to be relevant and meaningful across community organizations and sectors so that the right data could be obtained from those who needed it when needed. Such data, often originating from multiple sources and emerging from connections across sectors and systems,^5^ must enable shared situational awareness and problem‐focused decision making.^6^, ^7^

Local health care, public health, government, and community partners are uniquely positioned to respond to public health situations. Considering the need to accelerate vaccine distribution and delivery using a learning health system lens, situational awareness can be gained through maximal utilization of available data and insights into the local context.^7^ Such data and insights, combined with a deep understanding of community needs and appreciation of local capabilities and strengths, enables cross‐sector community partnerships to harness their assets and develop a powerful local distribution strategy. Building on trusting relationships with the local community, and in close alignment with the state, collaborations can use local knowledge and reliable learning methods to rapidly pursue equitable population immunity.^8^

**FIGURE 1:**
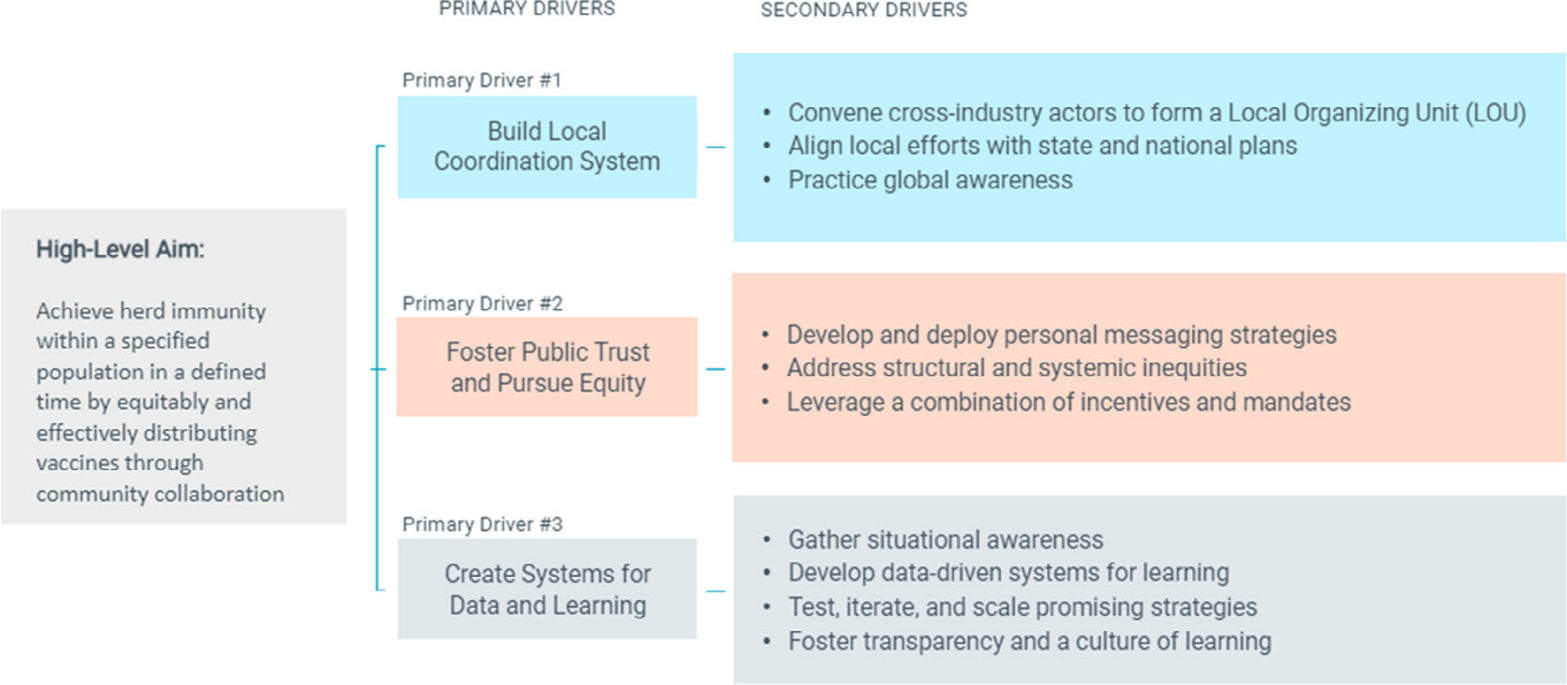
Phase 1 theory of change. Figure describes core drivers for theory of change.

Partners from the Institute for Healthcare Improvement (IHI), Civitas Networks for Health, Cincinnati Children's Hospital Medical Center, The Health Collaborative, Parkland Center for Clinical Innovation (PCCI), and Parkland Hospital in Dallas came together to learn from their collective experiences throughout the COVID‐19 pandemic. Learning centered on achieving a better understanding of how to attain a more comprehensive situational awareness of the population's COVID‐19 immunity. Partners believed that this awareness would drive local decision making and more equitable community‐wide protective responses. Based on the experiences of project partners and insights generated from vaccine delivery partnerships in metropolitan areas across the United States (Including Cincinnati, OH^9^; Dallas, TX^10^; Santa Cruz, CA^11^; region of Northeast, OH^12^), we developed a model to equitably achieve population immunity within a specified population in a defined time. The primary drivers of this theory of change included: (1) establish coordinated local testing and vaccine delivery systems; (2) foster public trust and pursue equity; and (3) use data to learn and scale successful strategies (Figure 1).

The present report details the second phase of this work, in which we aimed to learn more deeply from an expanded set of public health communities about their experiences with accessing and learning from data to inform decision making, including how their experiences might modify our initial theory. In addition, we sought to identify key supports and approaches through which community‐wide data and learning systems can be utilized to drive equitable public health decision making and put real‐time data in the hands of community leaders across sectors and of individuals within the community. The inquiry was framed by four broad questions related to the continuing evolution of COVID‐19 and its implications for potential future community‐wide public health crises:

1. How can we best simplify the collection, analysis, and use of data so that communities with varying degrees of experience and access to data can build situational awareness?
2. How can data be used to guide local decision‐making to decrease variation and improve safety and equity for all communities in a local geography?
3. How have communities answered these first two questions during COVID‐19 pandemic to‐date, and what are their needs and hopes for the future?
4. How can communities be more prepared for public health challenges sure to come?

## 2 METHODS

Semi‐structured qualitative interviews were conducted with 16 local health care, public health, government, and community partners. Participants were identified based on an interview rubric to have a distribution of participants across dimensions, including urban/rural settings, different regions of the country, and different levels of access to data. Specific participants who were interviewed by the Civitas team were then invited based on project partners' networks. The IHI team had no preexisting relationships prior to the commencement of this project. We included interviewees from both rural and urban settings, in a variety of geographic regions across the United States. To understand the range of experiences in data access and usage, we interviewed 10 leaders who worked at a state or county public health department and six who worked at state public health data exchanges (See Table 1). Some interviews were done individually while some were done in a group interview setting (noted in Table 1). Group interviews included multiple people from the same department and were conducted in the same manner as 1:1 interviews. The 16 leaders represented nine different organizations in seven different states, and their roles included Health Commissioner, Assistant Director/Deputy, Chief Program Officer, Chief Executive Officer directors of reporting and analytics. In addition to the interviews, we learned directly from the case study experiences of leaders in Cincinnati and Dallas, who created online tools and approaches throughout the various phases of the pandemic.

**TABLE 1:** Interviewee list. Complete list of interviewees organized by name, organization, and job title.

Number of Interviewees	State/Region	Organization
1	Ohio	Hamilton County Public Health Department
4	Texas	Houston Health Department
3	Texas	Tarrant County Public Health Department
1	Nebraska	CyncHealth
1	Oklahoma	Oklahoma City‐County Health Department
2	Maryland	Chesapeake Regional Information System for Patients (CRISP)
2	North Carolina	North Carolina Health Information Exchange Authority
2	Indiana	Indiana Health Information Exchange Authority
1	Texas	Dallas County Department of Health and Human Services

The interviews explored the four broad questions listed in the previous section, and also explored additional detail by probing to further understand the critical questions leaders needed to answer as they managed a community‐wide COVID‐19 response, how data informed those answers (including data sources used, operations, analysis, frequency of collection), what an “equitable response” meant to them, challenges faced in making data‐informed decisions, and structures and systems that enabled and/or hindered their response.

All interviews were conducted by a lead facilitator on Microsoft Teams, lasted between 45 and 60 min, and were recorded. Recordings were not transcribed, but extensive notes were taken during interviews on a Microsoft Forms sheet by a designated notetaker (see Table 2). When necessary, we referred back to the recordings for exact quotes or context. During the data analysis, four authors led the coding process by exporting the raw notes to an Excel spreadsheet and using deductive coding to identify themes and explore the relationship of those themes to our theory of change. The coders met weekly to discuss how they coded the interview notes and themes emerging from the interviews. Findings and themes were communicated back to the complete author list at bi‐weekly meetings for feedback and additional discussion. Interviewees provided consent to be identified in the table above as participating in the interviews. We confirmed confidentiality and identification with interviewees throughout the process.

**TABLE 2:** Microsoft forms note template. Complete list of interview questions and prompts in microsoft forms template.

Interviewee name	
Interviewer (person conducting the interview)	
Which dashboards or data sources have been/are most helpful to your community in managing the pandemic? How so/in what ways are you applying them?	[optional prompt if needed] For example, did you find value in the COVID‐19 dashboards published by Johns Hopkins, the New York Times, the CDC, or similar data published by state health departments? What organizations in the community depended on such data to carry out their mission? Are there unique data/analytics/programmatic things you developed locally that you found very useful? Are there things that you used during the pandemic that you have already started to expand into other areas/applications? ○ Is there a “wish list” for future applications?
How was data helpful in managing operations early in the pandemic?	For example, did it inform COVID‐19 testing, managing hospital capacity, vaccine distribution, reducing inequities? If so, how was the data used specifically? [potentially prompt if needed to clarify which data sources were used] Was further analysis required to apply the data? ○ How did you tailor the data to your local context? ○ Did you have data at various geographic scales (eg, street address, neighborhood, zip code, census tract, or county)? ▪ To what extent did organizations use hyperlocal data to try to decide hyperlocal decisions—e.g., use masks in this school, not that one And what does “hyper‐local” mean in your context? Is it relevant? What was your frequency of use (eg, daily, weekly, monthly)? ○ Did this cadence match the frequency of data update and publication? Who used these data? Who collaborated? Who were your go‐to partners for data? Did you face any challenges to access? ○ Was the data publicly available or was use governed by restrictions? How did you communicate data/insights to stakeholders, leaders, and the broader community (if at all)
What additional information would have been helpful to you at that early stage of the pandemic?	What operational needs had to be managed with limited or unavailable data? ○ What kind of information channels have been or are being built to manage these operations? ○ Looking back, what would you have liked to have done in the early‐stage response but could not because data or analyses were not available? ○ What data could have been more useful if collected and delivered at different geographic scales and frequencies? What kind of partnerships and approaches to collaboration would have been helpful in making data‐informed decisions and being more responsive to community needs? Would knowing local community immunity (vaccination‐acquired immunity + infection‐acquired immunity) be valuable and useful to your community?
Would knowing local community immunity (vaccination‐acquired immunity + infection‐acquired immunity) be valuable and useful to your community?	Are you taking your focus off COVID/turning it to other things re: your data collection and surveillance?
What is geographic area of responsibility of your organization? [Note: If we are in a shorter interview/crunched for time, could potentially ask about this in follow‐up]	What do you see as the most important socio‐demographic factors in this region for your COVID‐19 work? How was the pandemic response coordinated in your designated area? ○ Which body or group was responsible? ○ What structure supported the response?
Over the course of the pandemic, how did you ensure equitable response to community needs?	How did you identify and focus immunization efforts to support the most vulnerable members of your community? What information, resources, or tools would have helped you more effectively support this population? What strategies have you found most effective in building trust with the community?
Who else should we talk to understand your organizations and community's approach to managing the pandemic?	Examples of local entities that they might refer us to: public health departments, health care organizations, health improvement collaboratives, congregate care groups, emergency management agencies, or political offices
[If time], is there anything else you would like to share or wish I would have asked?	

This work was undertaken to understand and improve ongoing activities in public health settings, and not to produce generalizable knowledge. As such, it constituted operational improvement activities that are exempt from ethics review. The primary purpose of this report is to share lessons learned from the experiences in these specific settings.

During data analysis, we exported the raw notes to an excel spreadsheet and used deductive coding^13^ to identify themes manually and explore the relationship of those themes to our theory of change. We used both interviews and case studies from our partners to learn more deeply about our theory of change. Understanding the partners' experience was critical to drafting our theory of change, and we utilized the interviews to provide a broader perspective to reinforce and/or challenge the identified drivers in the initial driver diagram.

## 3 RESULTS

### 3.1 Results from interviews with public health leaders

Our initial theory of change identified the following primary drivers: build local coordination system, foster public trust and pursue equity, and create systems for data and learning. The interviews provided insights into these drivers, which added to our initial theory of change, including insights related to equitable vaccine coverage. Interviews also revealed new themes regarding an equitable, community‐based approach to the current phases of COVID‐19 and other public health emergencies. The learning that emerged from the interviews led to an adapted theory of change that is inclusive of both the initial primary drivers and the themes identified in the interviews. The four key themes that were identified in the interviews include (Table 3 includes both the themes as well as sub‐themes):

1. Fostering a foundation of trust and pursuing equity;
2. Creating cohesive community coalitions with shared alignment and goals;
3. Using accessible, real‐time data to create systems for data and learning; and
4. Creating adaptable, resilient infrastructure to create systems for data and learning.

**TABLE 3:** Summary of themes from interviews with public health leaders.

Key theme from interviews	Connection to initial theory of change	Sub‐themes
Fostering a foundation of trust and pursuing equity	Reinforces and adds additional considerations for primary driver 2 Highlights connection across all 3 primary drivers	Relationships between actors and the community Political alignment, including public health messaging and recommendations Tension of keeping individual level data private, while transparently sharing community data Investing in the community: hiring locally Using data to identify areas with the most need (highest COVID‐19 case rates, lowest vaccine rates) The technical elements that build or undermine trust
Creating cohesive community coalitions with shared alignment and goals	Necessary to achieve the aim relevant within each primary driver	Data and progress sharing between partners Regular round table touch points to share progress/challenges Engagement of community liaisons, leaders, and champions Cross‐agency relationships, including political, health, and multi‐sectoral partners Accessible sharing between systems (state and local, rural, and urban)
Using accessible, real‐time data to create systems for data and learning	Brings to life primary driver 3 in specific ways	Disaggregating data by race, socioeconomic status Alignment in reporting specimen vs reported date Timeliness in reporting contributed to trust Transparency in reporting contributed to trust Privacy and transparency (including HIPAA and legal barriers, and at different levels)
Creating adaptable, resilient infrastructure to create systems for data and learning	New wording for primary driver 3	Repurposing sites for testing and vaccine (and future needs) Mobile clinics used to target specific areas within a community The physical elements that build or undermine trust Systems in relation to how data is collected and shared, and how models are organized Creating systems that help smaller organizations and clinics Leveraging technology used for COVID in service of chronic disease management

There were significant tensions (and sometimes contradictions) related to data sharing, cultivating trust with community members, and supporting an equitable response to COVID‐19. We describe these tensions below.

The first theme related to **fostering public** trust and related to the initial theory of change. This theme validated the importance of primary driver 2 (“Foster public trust and pursue equity”) and added additional considerations for how that driver comes to life. This theme also highlighted the interconnection across all three primary drivers, as public trust and equity are essential for building and supporting a local coordination system and creating adaptable systems for data, infrastructure, and learning.

Interviewees shared how fostering public trust required a balance between maintaining data privacy at the individual level and reporting transparently at the community level with enough specificity/granularity to support actions being taken. When actors used data to focus on locations within their geography with the highest vaccine need (across interviewees, communities of focus included people experiencing poverty, communities of color, and older adults), they were able to build trust within those communities. However, multiple interviewees cited that further disaggregation of COVD‐19 data (eg, at the level of census tracts, or zip codes) would have been helpful in order to identify pockets of populations with highest need. Even then, data at the zip code or census tract level might not have been sufficiently granular to make neighborhood‐level decisions. In addition, data that were undermined by technical challenges, such as delayed reporting, resulted in a decrease in public trust of the localized COVID‐19 response. One interviewee described having to take additional steps to report on COVID‐19 cases to work around barriers, such as laws that limited disclosure of COVID‐19 testing results to anyone but the provider who ordered the test. This created blind spots in a patient's care and demonstrated how some state regulations had not caught up with the realities of public health at this point in the pandemic.

The interviewees also described partnering with trusted community leaders to share key messages as a means of fostering trust and pursuing equity. Alignment between local health care, public health, government, and community partners around public health messaging and recommendations, such as masking and social distancing, facilitated trust between public health departments and the community. In areas where messaging did not align, interviewees reported more difficulty in implementing community‐wide public health measures.

The second theme related to creating cohesive community coalitions with shared alignment and goals. Cohesive community coalitions were a necessary component to achieve the aim relevant within each primary driver.

Although **coalition building** is not new, the COVID‐19 pandemic has provided a major threat to the health of communities, resulting in an unprecedented alignment of goals between stakeholders. Public health teams that did not report a positive relationship with other actors (such as disagreements on COVID‐19 masking requirements or social distancing recommendations) in their community were more likely to discuss challenges in responding to the COVID‐19 pandemic. These challenges included an inability to engage key stakeholders, difficulty managing competing priorities, and disagreement on how data were collected and shared. Another interviewee referenced how their partnership with Medicaid improved during the pandemic, as the collaboration provided access to ongoing data to monitor the local Medicaid population, understand where the needs were, and deploy resources accordingly. Teams that did have positive relationships with their community, state leadership, and health care systems were more likely to share their successes and future applications from learnings of this pandemic. For example, partnering with sewage departments supported the regular testing of wastewater, which enabled monitoring of infection rates. Partnership with the education department was crucial to enable regular review of the safety measures in schools and classrooms. Partnership with convention centers and private businesses was critical to setting up mass testing and vaccine administration sites. Interviewees also described successes when they directly engaged community members, either by hiring local leaders to positions of power or creating equity alliance councils. In both cases, partnership and engagement was designed to continue during future COVID‐19 phases and, potentially, future public health challenges.

Our third theme related to having access to real‐time data to facilitate data and learning. This theme reinforced and brought to life our third primary driver, with the goal of having adaptable systems for data and learning.

All interviewed local health officials described a lack of **accessible, real‐time data** and cited delays in reporting as a significant challenge to their pandemic response. One interviewee described having to share data with the state, who shared it with the CDC, and the CDC reported it back to the state—a time‐consuming information loop that served only the purpose of being allowed to report the data publicly. Another challenge was the backlog in processing of laboratory data, which in turn delayed public health departments efforts to report to the public. In many cases, the fast‐moving public health situation had evolved by the time data were able to be shared with the public. This resulted in community members feeling that the recommendations were outdated or, in some cases, intentionally hidden, both of which had a direct impact on the public's trust in the county's response. In addition, there was also a lack of agreement and alignment from the local to state level regarding how data were reported—whether based on the date the specimen was collected or the date the results were reported. This resulted in further opportunities to erode community trust. Conversely, some interviewees indicated that the pandemic response provided “the best population level data that we've ever been able to receive.” One interviewee described how they were using a technology solution designed for customer relationship management for both contact tracing and reconciliation of data from multiple centers. The use of available technologies also connects to the prior theme of coalition building, as in many cases, technology was used to mitigate challenges and support analysis and communication. For example, one interviewee used online visualization software to resource vaccine clinics and said it filled a gap by “creating a line of communication between public health and health care that was previously missing.” Public health departments also reported working on applying these technologies to address other preparedness shortcomings and future public health challenges.

Our fourth theme related to creating adaptable, resilient infrastructures to create systems for learning, further contributing to our third primary driver. It reinforced and added a key element of not only having systems that are adaptable within a virtual environment, but also ones that include physical resources and the built environment.

Interviewees noted that adaptability and repurposing systems are important in terms of the **physical resources, built, and virtual environments.** For example, repurposing sites used for COVID‐19 testing to administer vaccines presented differing challenges such as accessibility for those who drop up, used public transportation, or walked. Storage often requires that the vaccine be maintained at the correct temperature during administration. Interviewees used mobile clinics to target hard‐to‐reach populations, confirming that being able to repurpose and reposition sites has the potential to address not only COVID‐19 but future threats to public health.

Virtual data systems that are adaptable by different stakeholders arose as a necessity for future public health interventions. It was challenging to retrieve data from various sources such as the government, public health, and health care data systems that did not communicate with each other. One interviewee described how small rural clinics in their district could not access the portal in the large health care system due to technological limitations. This person described how they adapted the portal, making it somewhat “less sophisticated” but more easily shared, making data more accessible for these clinics. While interviewees acknowledged data sharing across actors had the potential for HIPAA and data privacy violations, they also described robust onboarding processes to offset these risks. This allowed key actors to use data for decision making across the public health field, as long as the data shared between public health and health care actors were not publicly available. The more widely accessible the data were, the larger potential there was for a timely, effective, equitable response to COVID‐19. Table 4 provides illustrative quotes from each of the key themes identified in the interviews.

**TABLE 4:** Illustrative quotes from interviewees.

Key theme	Illustrative quote
Fostering a foundation of trust and pursuing equity	“At different moments I'd be challenged by either reporters or the public. I was often challenged about small sample size (which you cannot be transparent because of the HIPAA line) and do not want to identify. Ethical considerations with the data made it difficult.”
	“Engaging local community leaders by use or social media tools and engaging respectable doctors or academic partners with linkages in the community to try and get ahead of trust issues… sometimes people do not trust government but trust community leaders.”
Creating cohesive community coalitions with shared alignment and goals	Having all the public health leaders access the data and disseminate the information was key. They had twice a week COVID calls which… were incredibly well‐received and attended. Included everyone from nursing homes to meat packing plants, and people shared ideas to support or control [COVID‐19].”
Using accessible, real‐time data to create systems for data and learning	“We've developed a lot of ‘state of the art tools’ (for future application) where we'd send text messages out. We have these trigger codes for reportable conditions and are working on chronic disease trigger codes to help get more real time information from hospitals on this data.”
	“We used numbers to push masking and vaccinating when numbers were getting high… Data could help predict surges and valleys between 10 to 14 days in advance. If the systems broke down and data got backed up, it could be a case dump of weeks. Real time means within 2–3 days old, at most.”
Creating adaptable, resilient infrastructure to create systems for data and learning	“Rural hospital systems needed a more simplified, easier to use and access dashboard which is what IHIE created. This will now allow us to give access to local health departments and smaller settings.”

## 4 RESULTS FROM PARTNERS' EXPERIENCES IN DALLAS COUNTY AND CINCINNATI

The experiences described below were both foundational in partners' development of the original theory of change, and, as the pandemic progressed, the continued experiences in these geographies reinforced elements of the primary drivers in that theory.

As the first COVID‐19 cases were being reported across the United States, partners in Cincinnati identified a need for meaningful situational awareness. The resulting regional response benefited from cross‐sector partnerships, data sharing, and collaboration that have been described in depth elsewhere.^6^, ^7^ As an example, the Cincinnati team employed a range of methods that were applied to consider how and where to locate testing and vaccine sites to maximize regional coverage. More specifically, the team mapped all existing sites (eg, hospitals). They did overlays to characterize access (eg, bus lines, drive time, walk time) as well as overlays to inform responses that were equitable (eg, census tract‐level socioeconomic deprivation index). These overlays highlighted gaps in coverage. The team then identified where new sites could be located to overcome these gaps. With pins placed on these virtual maps, community engagement teams worked with local community leaders to identify sites that were accessible, trusted, and open to being set up as testing (or vaccine) sites near those pins. These included recreation centers, libraries, and community organizations like YMCAs. These methods—robust situational awareness, merging advanced analytics with deep community engagement—have enabled a more optimal set of responses to COVID‐19. They are now being adapted to other population health challenges (eg, inequities in the experience of childhood asthma, food insecurity, child abuse and neglect).

Dallas County Health and Human Services led all their local COVID‐19 initiatives in direct collaboration with Parkland Center Clinical Innovation (PCCI), Parkland Health and in alignment with all local municipalities, health providers, and community leaders. The coalition took a hyper‐localized, data‐driven approach to guide local interventions and coordinate efforts. Technology‐based solutions were developed and deployed to identify people most at risk of exposure to the virus and subsequently those with limited or diminished immunity. PCCI developed a Proximity Index and a Vulnerability Index to help identify, educate, and care for the county's high‐risk population. Person‐level data were used to identify individual risk and vulnerability for direct to patient outreach, contact tracing, and personal communication. Aggregate census block‐level data were used to target education and outreach (in a culturally sensitive manner based on local ethnic and racial demographics) as well as placement of mobile and pop‐up testing and vaccination sites.^14^ Via a newly developed community protection algorithm and dashboard, the coalition of partners continue to track the ever changing local and national dynamics to anticipate community risk, guide vaccinations, and anticipate magnitude and impact of emerging waves.

**FIGURE 2:**
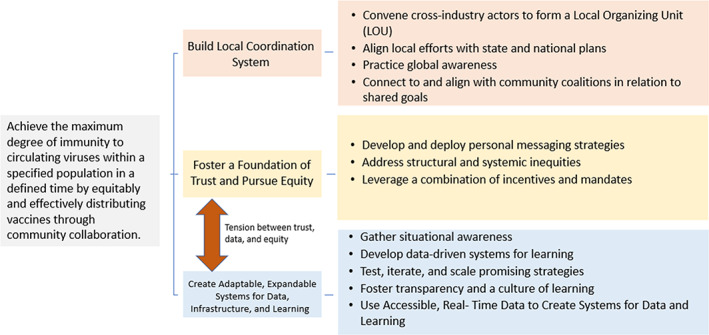
Revised theory of change. Figure below depicts the revised theory of change.

The Cincinnati and Dallas experiences reinforced the three primary drivers in the theory of change. What also became quite clear as the pandemic unfolded was that the initial aim in the theory framed as “achieving herd‐level immunity” was quite unlikely, especially given the increasingly more charged political nature of the pandemic and vaccination and the rapid evolution of the virus. Thus, we reframed the aim in the theory of change to be (with new wording in italics) to achieve *the maximum degree of immunity to circulating viruses* within a specified population in a defined time by equitably and effectively distributing vaccines through community collaboration (Figure 2).

## 5 DISCUSSION

The COVID‐19 pandemic provided a major challenge to public health services and an unprecedented opportunity for collaboration and innovation across health care organizations, public health departments, civic structures, local businesses, and the communities they serve. Our qualitative study supports our initial prediction that close collaboration between the leaders of public health, health care, government, and community organizations would accelerate the speed and effectiveness of the required responses to the pandemic in specific communities, and that availability of data to track local variation in infections and the vaccination efforts in those communities is essential for planning and implementation of that response.^15^ Our survey also confirmed the critically important role of trust in driving effective partnerships and community behaviors, and the role of data transparency and true collaboration with communities in building (or undermining, when absent) that trust.

We focused on core elements of a public health emergency awareness, learning, and response system that often did not exist before the pandemic. The respondents we interviewed led a variety of local organizations that were constituents of newly formed or newly expanded local partnerships, driven by a common purpose to support their local communities' efforts to manage various phases of the COVID‐19 pandemic. The resulting collaborations and commitment to supporting communities with transparent plans and data engendered trust between partners and between the partners and the communities they serve.

The interviews point to the crucial need for timely data on infections in a pandemic fueled by an evolving virus with constantly changing infectivity and virulence. The leaders we surveyed highlighted the challenges of acquiring and using multisource data for pandemic surveillance and responses such as testing and vaccination, as well as the barriers to making data transparently available to stakeholders, that delayed decision making and eroded community trust.

The opportunity to present data aggregated from multiple sources in a condensed format that was optimally designed to inform COVID‐19 related decision‐making by local multi‐partner collaborations was illustrated by the data dashboard created by Cincinnati Children's Hospital Medical Center (https://www.cctst.org/covid19). This dashboard provided a range of key data elements that described both the progress of the disease and the community response to the pandemic, as well as real‐time, local level to understand the burden of disease in specific communities in a way that could inform focused intervention and equitable response. Using principles from common and special cause variation resulted in the display of data were displayed as line graphs over time in a format that differentiated normal variation in data from significant deviances from expected (based on principles of statistical process control, which have been applied to a broad range of health care contexts, including specifically to COVID‐19).^16^, ^17^ This allowed for more timely identification of changes in pandemic trajectory and the corresponding interventions from the coalition.

**FIGURE 3:**
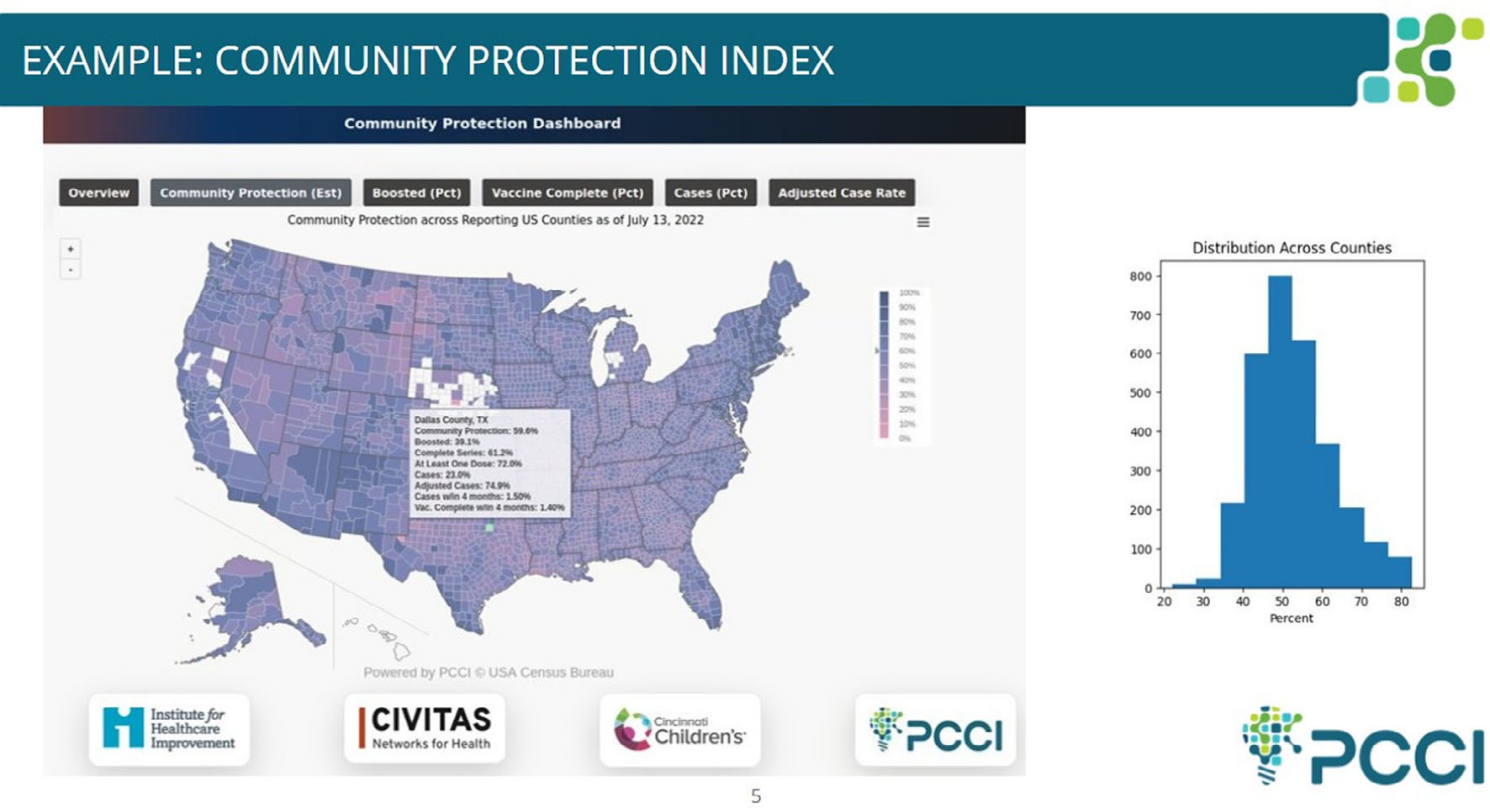
Screenshot from Community Protection Dashboard, available at https://www.civitasforhealth.org/community‐protection‐dashboard

While our inquiry focused on the use of data primarily by public health organizations, the public, too, needs and expects clear and credible information they can use to make informed decisions about public health issues like COVID‐19.^18^, ^19^ The interviews we conducted highlight this need, especially when thinking about responding equitably to public health crises and keeping those most impacted at the forefront. To respond to this need, partners created a tool—the Community Protection Dashboard^6^ to make publicly available COVID‐19 data more accessible and usable to individuals in counties across the United States (Figure 3). The Dashboard was developed by PCCI and informed by a joint effort between the partners of this work. The PCCI tool provides an aggregate estimate of local immunity based on vaccine uptake and prior infections with COVID‐19 and was intended to provide better situational awareness for both the communities themselves and the entities charged with caring for them. The dashboard offers periodically updated local dynamic vulnerability awareness at the county level, national contextualization, and the ability to identify emerging trends and forecast impacts based on cross‐region comparisons. The data could be used by individuals to make decisions (eg, where to go, whether to wear a mask) that conform to their level of comfort, and administrators can use the dashboard to make decisions on activities and interventions. The data could also be used by administrators who can make decisions on policy and resource deployment.

The activity and analyses described in this Experience Report have several limitations. While we interviewed 16 public health and health information exchange leaders working in geographically and demographically diverse areas of the United States, areas and populations not represented by interviewees may have distinct needs potentially not overlapping with what was reported by respondents. The sampling and number of respondents was determined by time constraints and somewhat reliant on existing relationships. Findings may have been richer if additional interview time had been available and/or if resources permitted interviews with additional leaders. Ultimately, our goal in qualitative interviews was to understand the depth of experience in the sample we had, which is why interviews were done instead of a broader survey. We developed a driver diagram well before the interview guide was developed, so that interview questions may have been influenced by our working hypothesis of data needs and barriers. However, potential confirmation bias was controlled by having individuals not familiar with the driver diagram or involved in the community work developing the guide, administering the interviews, and analyzing the resulting data.

Nonetheless, the inquiry we undertook is relevant to the ongoing and future management of COVID‐19 and future pandemics. These approaches (local collaboration and planning, data coordination and transparency, building trust in the community) are also likely applicable to several existing and urgent public health and community health care needs. Apart from application to related episodic community infection (eg, influenza), this approach can be imagined for addressing common community‐influenced chronic conditions like childhood asthma and deeply location dependent public health issues like food insecurity, and homelessness.

## CONFLICT OF INTEREST STATEMENT

The authors declare they have no conflicts of interest and nothing to disclose.
